# Reduced Germination of *Orobanche cumana* Seeds in the Presence of Arbuscular Mycorrhizal Fungi or Their Exudates

**DOI:** 10.1371/journal.pone.0049273

**Published:** 2012-11-07

**Authors:** Johann Louarn, Francis Carbonne, Philippe Delavault, Guillaume Bécard, Soizic Rochange

**Affiliations:** 1 Université de Toulouse; UPS; UMR 5546, Laboratoire de Recherche en Sciences Végétales; BP 42617 Auzeville, F-31326 Castanet-Tolosan, France; 2 CNRS; UMR 5546; BP 42617, F-31326 Castanet-Tolosan, France; 3 Laboratoire de Biologie et Pathologie Végétales, SFR 4207 QUASAV, LUNAM University, Nantes, France; Ghent University, Belgium

## Abstract

Broomrapes (*Orobanche* and *Phelipanche* spp) are parasitic plants responsible for important crop losses, and efficient procedures to control these pests are scarce. Biological control is one of the possible strategies to tackle these pests. Arbuscular Mycorrhizal (AM) fungi are widespread soil microorganisms that live symbiotically with the roots of most plant species, and they have already been tested on sorghum for their ability to reduce infestation by witchweeds, another kind of parasitic plants. In this work AM fungi were evaluated as potential biocontrol agents against *Orobanche cumana*, a broomrape species that specifically attacks sunflower. When inoculated simultaneously with *O. cumana* seeds, AM fungi could offer a moderate level of protection against the broomrape. Interestingly, this protection did not only rely on a reduced production of parasitic seed germination stimulants, as was proposed in previous studies. Rather, mycorrhizal root exudates had a negative impact on the germination of *O. cumana* induced by germination stimulants. A similar effect could be obtained with AM spore exudates, establishing the fungal origin of at least part of the active compounds. Together, our results demonstrate that AM fungi themselves can lead to a reduced rate of parasitic seed germination, in addition to possible effects mediated by the mycorrhizal plant. Combined with the other benefits of AM symbiosis, these effects make AM fungi an attractive option for biological control of *O. cumana*.

## Introduction

Broomrapes (*Orobanche* and *Phelipanche* spp) and witchweeds (*Striga* spp) are parasitic plants from the *Orobanchaceae* family. They attach to the roots of host plants from which they drain water, mineral and photoassimilate resources. Together, *Orobanchaceae* species can attack a wide range of host plants, and those that infect crops can cause considerable agricultural damage. In Africa, *Striga* spp represent a major and increasing threat to cereal crops, affecting millions of farmers [1]. Broomrapes are of particular concern around the Mediterranean basin and in Eastern Europe, where they attack a large number of crops including *Solanaceae*, *Fabaceae*, *Brassicaceae* and *Asteraceae*. In this study we focus on a broomrape species that became weedy only recently, *Orobanche cumana*. Unlike other broomrape species, *O. cumana* exhibits a restricted host range and essentially attacks sunflower (*Helianthus annuus* L.) [2].

As obligate parasites, broomrape seedlings can only survive for a few days after germination before connecting to a host root. The germination of their seeds has evolved to become dependent on compounds secreted by host roots, and thus occurs only in the favourable presence of a host. Germination stimulants have been identified in several host plants [3]. Broadly speaking, the most potent are strigolactones, a family of carotenoid-derived metabolites, but germination stimulants have also been identified in several other classes of metabolites [4]. Although sunflower produces strigolactones [5], dehydrocostus lactone (DCL) has been proposed as a major germination stimulant in this species [6].

When stimulated after a conditioning period of several days under appropriate temperature and humidity, seeds germinate and produce a radicle that can adhere to a host root. A haustorium is formed, which allows the penetration into the cortex and the exploitation of the host’s resources through the development of vascular connections. From this stage, *Orobanchaceae* engage in their truly parasitic lifestyle [7]. A new organ called the tubercle develops on the host root and acts as a strong sink to accumulate reserves [8]. Later, a stem arises from the tubercle, and emerges above ground to produce an inflorescence, giving rise to thousands of new seeds able to survive in soil for many years [9].

Research efforts have aimed to identify resistance mechanisms in host plants. Tolerant or resistant genotypes have been identified in a number of hosts of *Orobanche spp*, and in some cases the physiological basis for improved resistance has been characterized. Such mechanisms can act before or after root penetration and include a lower production of germination stimulants, the production of germination inhibitors, the formation of physical barriers against the parasite, the release of compounds inducing tubercle necrosis, the occlusion of vascular connections between host root and parasite, and a low supply of carbon to the parasite through competition with the host plant carbon sinks [10]–[12]. From a genetic point of view, resistance often shows a complex heritability [13]. The *O. cumana*/sunflower interaction is an exception in this context, since the existence of several dominant resistance genes has been demonstrated (e.g. [14]). Unfortunately such sources of resistance can rapidly be overcome by the parasite, and new *O. cumana* races appeared that could attack the successive “resistant” sunflower genotypes [15]. This underlines the need for approaches that favour the intervention of several physiological processes and genes.

Biological control is one of several alternative strategies to tackle the parasitic weed problem. For example, *Fusarium* spp have been used against *O. cumana* and *P. aegyptiaca* [16]. Among additional potential control agents, Arbuscular Mycorrhizal (AM) fungi could be of particular interest. These fungi live in all kinds of terrestrial ecosystems and associate with the vast majority of land plants, forming the most widespread type of symbiosis on Earth [17]. In exchange for hexoses, AM fungi can supply their host plant with water and minerals, and provide a certain degree of protection against pathogens [18]. Like parasitic weeds, AM fungi are obligate biotrophs and rely on stimulation by their host to engage in their developmental cycle and colonize a plant root. Remarkably, they were shown to respond to the same chemical cues as the majority of *Orobanchaceae* seeds: early fungal development and metabolism are strongly stimulated by strigolactones [19], [20]. Because of the reported protective effect of AM fungi against various pathogens, their influence on *Orobanchaceae*/host interactions was investigated. Lendzemo & Kuyper [21] first showed that mycorrhizal inoculation could alleviate the damage caused on sorghum by *Striga hermonthica*, and in some cases reduce the level of host infestation. This effect was later attributed to a reduced release of germination stimulants by mycorrhizal plants [22], [23]. Mycorrhizal colonization of pea plants was also shown to reduce the production of *Orobanche* and *Phelipanche* seed germination stimulants [24]. In tomato, Lopez-Raez et al. [25] demonstrated biochemically that root colonization by AM fungi decreased the synthesis and exudation of strigolactones.

The aim of the present study was to investigate whether, and how, AM fungi affect the *O. cumana*/sunflower interaction. Root colonization by AM fungi was found to confer some degree of protection against *O. cumana*. This effect seemed to involve a lower production of germination stimulants, but also an antagonism between AM fungi and the parasite: the presence of fungal spores or fungal exudates alone could reduce the seed germination of *O.* *cumana*. Therefore, the effects of AM fungi on the pathosystem sunflower/*O. cumana* seem to be more complex than previously anticipated.

## Materials and Methods

### Plant and Fungal Materials

Sunflower (*Helianthus annuus L.*) seeds of the broomrape-susceptible variety 2603 were provided by Syngenta Seeds (Toulouse, France). Seeds of *Orobanche cumana* (Wallr.) race E were collected near Ecija, Spain, and provided by Dr B. Perez-Vich. Seeds of *Phelipanche ramosa* (Pomel) pathovar C were collected in France (Saint Jean d’Angely, 2005). Seeds of *Striga hermonthica* were collected in Sudan in 1999. No specific permits were required. The locations where seeds were collected were not privately-owned or protected in any way, and these field studies did not involve endangered or protected species.

Sterile spores of *Rhizophagus irregularis* (formerly *Glomus intraradices*, DAOM197198) produced *in vitro* on root organ cultures were purchased from Agronutrition (Carbonne, France). Spores of *Gigaspora rosea* (DAOM194757) were produced in co-culture with leek and surface-sterilized as described in [26].

### Plant Growth Conditions

Sunflower seeds were sown in 250-mL pots containing sterilized charred clay (Oil Dri, Klasmann, France) as an inert substrate, and kept in a growth chamber under a 16 h photoperiod (22°C day, 20°C night). Plants were watered daily with a low-phosphate half-strength Long Ashton Nutrient Solution (LANS, [27]) containing a final concentration of sodium dihydrogen phosphate of 7.5 µM. This fertilization regime promoted root colonization by AM fungi [28].

For mycorrhizal inoculation, 1,000 sterile spores of *R. irregularis* per pot were mixed with the substrate. Root colonization by AM fungi was determined using the gridline intersect method [29], after staining roots with Schaeffer black ink [30]. For the assessment of resistance to *O. cumana*, the substrate was mixed with *O. cumana* seeds (approx. 1,250 seeds per pot), with or without AM fungal spores (1,000 spores per pot). The pots were left for seven days under moist conditions in the dark (16 h at 22°C and 8 h at 20°C), to promote conditioning of *Orobanche* seeds. Then, sunflower seeds were sown directly in the pots. Infection by *O.* *cumana* was determined after five or six weeks in culture by counting the number of tubercles.

### Preparation of Root Exudates

Four-week-old plants grown as described above were removed from the substrate. The roots were rinsed and incubated in low-phosphate LANS for 24 h at room temperature (roots were not separated from the aerial parts). Crude root exudates were filtered, and root dry weight was determined.

### Preparation of Exudates of AM Fungi

For each replicate 10,000 spores of *R. irregularis* or 2,000 spores of *G. rosea* were suspended in 10 mL sterile water in 55 mm Petri dishes (a smaller number of spores was used for *G. rosea* because they are larger than those of *R. irregularis*). They were incubated in the dark at 30°C under 2% CO_2_ for seven days. Most spores germinated during this period. The suspension was filtered through a 0.22 µm filter and the filtrate constituted the "1X" germinated spore exudates. This solution was either used directly in seed germination assays, or diluted in water to 0.1X or 0.01X before use.

### Orobanchaceae Seed Germination Assays

Germination assays were conducted as described in [6] with minor modifications. Broomrape seeds were surface sterilized by treatment with 2.6% NaClO for 5 min, then rinsed five times with sterile water. For each replicate, around 100 seeds were sown on a 25 mm glass-fiber filter (Whatman GF/F) moistened with 500 µL sterile water, in a 35 mm Petri dish. The Petri dishes were sealed with parafilm and incubated in the dark at 22°C for 11 days. After this conditioning period, excess water was removed from the glass fiber filter. Seeds were treated with 500 µL solution (see below), and incubated in the dark at 22°C for another eight days before germinated seeds were counted using a stereo microscope. For *Striga hermonthica*, seeds were incubated at 35°C throughout the test.

Control batches were treated with 10^−8^ M GR24 in 0.1% acetone (positive control) or 0.1% acetone alone (negative control). Crude sunflower root exudates were diluted so that 1 mL of solution contained the exudates produced by the equivalent of 4 mg root dry weight. For the experiment described in Fig. 4 and Fig. 5, *O. cumana* seeds were treated with AM germinated spore exudates prepared as described above. Alternatively, ten AM fungal spores were deposited on the glass-fiber filters together with *O. cumana* seeds.

### Arabidopsis and Leek Seed Germination Assays

Seeds were surface-sterilized by treatment with 2.6% NaClO for 5 min. They were then rinsed three times with sterile water, incubated in ethanol for 2 min and allowed to dry. For each replicate, 50 seeds were sown on M medium [26] solidified with 4 g/L Phytagel (Sigma) in two-compartment Petri dishes, and left at 4°C for 48 h before treatment. Seeds in each compartment were treated either with 1 mL water (control) or with 1 mL of 1X AM spore exudates obtained as described above. Seeds were incubated at 25°C under a 16 h photoperiod for two days for *A. thaliana* and 10 days for leek, before germination rates were determined.

### Seed Viability Test

2,3,5-Triphenyl Tetrazolium Chloride (TTC) viability staining was performed as described in [31] with minor modifications. Eight days after treatment with *R. irregularis* spore exudates, broomrape seeds were treated with a 1% solution of TTC in water, and the Petri dishes were sealed and incubated in the dark at 30°C for 24 h. Seeds were considered as viable if they displayed a pink/red colour.

### Gigaspora Rosea Hyphal Branching Bioassay

Crude root exudates were extracted with 1 volume of ethyl acetate as described in [28], then dried and resuspended in 500 µL of 10% acetonitrile per 60 mg root dry weight.

Hyphal branching bioassays were carried out as described in [20]. Briefly, germinated hyphae were treated with 10 µL root exudate extracts. Newly formed hyphal apices were counted 48 h after treatment.

### Mass Spectrometry Analysis of Strigolactones

Root exudates were extracted and analysed by LC-MS/MS in the Multiple Reaction Monitoring (MRM) mode as described in [28]. Transitions corresponding to orobanchyl acetate and 5-deoxystrigol (353>256 *m/z* and 411>254 *m/z*, respectively) were monitored.

### Mass Spectrometry Analysis of DCL

Root exudates were extracted using Solid Phase Extraction tubes (Oasis® HLB 6cc, Waters). The tubes were pre-conditioned with 3 mL of methanol followed by 3 mL ultra-pure water. The samples were loaded on the column, then eluted with 2×3 mL acetone and dried under gaseous nitrogen flow.

Root extracts were obtained as follows: roots frozen in liquid nitrogen were ground using a mortar and pestle and 15 g of powder were resuspended in 100 mL water. This solution was mixed to 100 mL dichloromethane for 5 min. The mix was then filtered under vacuum on a GF/F filter (Whatman) to eliminate remaining solid particles. The organic phase was collected and dried under gaseous nitrogen flow.

The solid residue of root exudates or root extracts was resuspended in acetonitrile:hexane (1∶1; v:v). Samples were analyzed by GC-MS using a GC Trace gas chromatograph coupled with a TSQ Quantum triple quadrupole mass spectrometer (Thermo Scientific), with splitless injection at 230°C, and electron impact ionization at 70 eV with a source temperature of 250°C. Chromatographic separation was achieved on a ZB-5MS capillary column (30 m×0.25 µm×0.25 mm, Phenomenex), using helium as a carrier gas at a flow rate of 1 mL/min. The oven temperature was initially held at 60°C for 2 min, then increased to 340°C at a rate of 12°C/min. Retention times and ion masses were compared to those of a DCL standard (Sigma).

### Statistical Analyses

Analysis of variance (ANOVA), or Student’s t-test in the case of a two-sample comparison, were performed when the homoscedasticity and normality criteria were met. In the other cases, i.e. for the *O. cumana* seed germination assays where n = 5, non-parametric tests were used: the Wilcoxon test for two-sample comparisons, or the Kruskal-Wallis test for multiple comparisons [32]. All statistical analyses were performed with R version 2.12.1 (http://www.r-project.org/) using the pgirmess package.

## Results

### Mycorrhization Moderately Protects Sunflower against *O. cumana*

To investigate whether mycorrhizal fungi can offer a protection against *O. cumana* in sunflower as they do against *Striga* in sorghum, sunflower seedlings were inoculated simultaneously with spores of the AM fungus *Rhizophagus irregularis* (formerly *Glomus intraradices,* [33]) and seeds of *O. cumana*. Experimental conditions were designed to determine as clearly as possible the effect of mycorrhizal inoculation (see Materials and Methods). The root systems of all plants were examined. AM fungal structures were never observed in non-inoculated control plants, while root colonization levels in AM-inoculated plants ranged from 25 to 70% depending on experiments.

**Figure 1:**
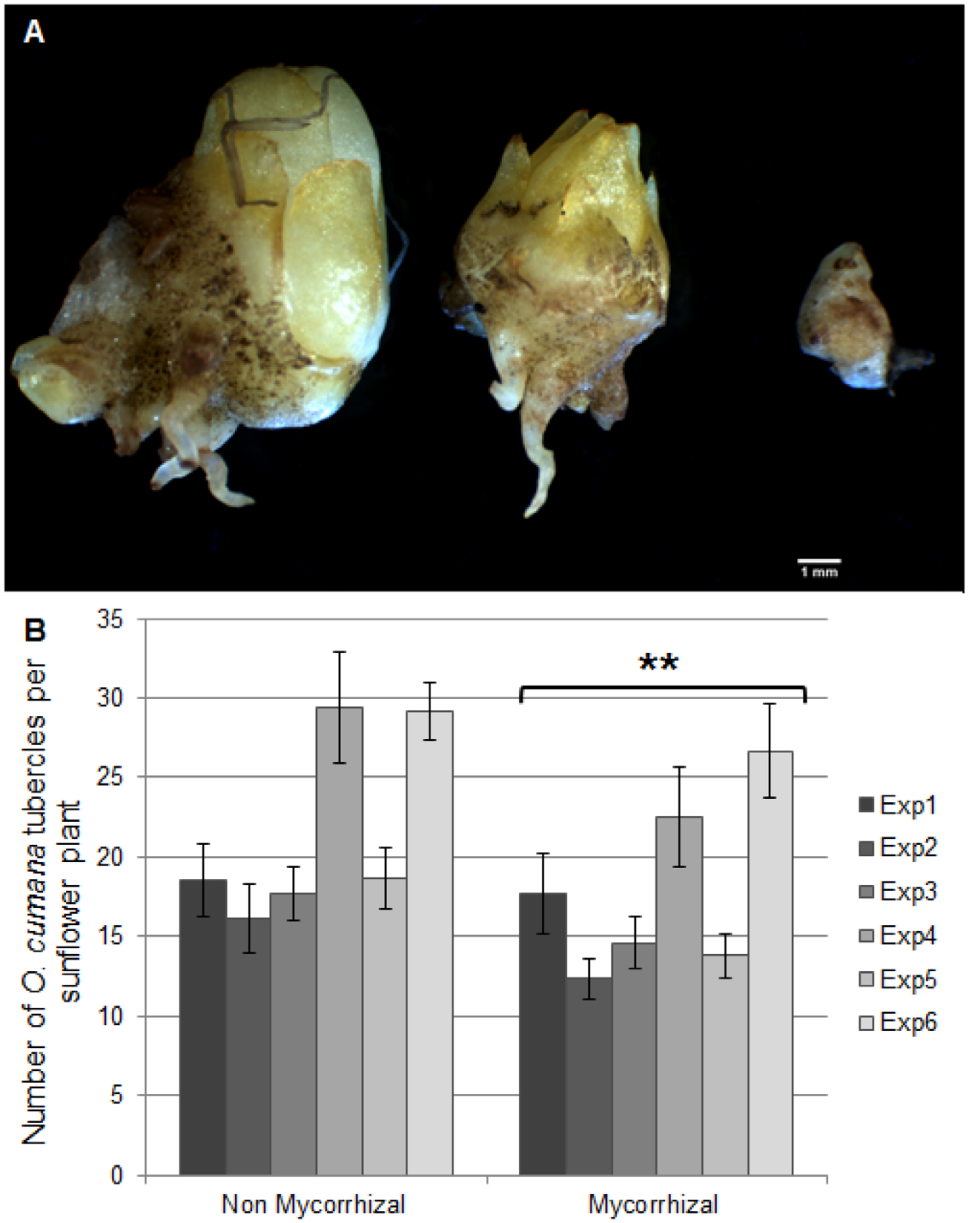
Effect of mycorrhizal status on sunflower infestation by *O. cumana*. Sunflower plants were inoculated with *O. cumana* seeds, in the presence or absence of AM fungal spores. A- Photograph of *O. cumana* tubercles of different sizes observed on sunflower roots. At the time of harvest (five or six weeks post inoculation) almost all attached broomrapes were at the tubercle stage. B- Number of *O. cumana* tubercles. The results of six independent experiments (Exp1 to Exp6) are presented. Values represent the average number of *O. cumana* tubercles per plant, +/− s.e.m (n = 8 to 10 plants per condition for each experiment). The asterisks indicate a highly significant effect of mycorrhizal inoculation according to multi-way ANOVA (P = 0.008, see also Table S1).

The tubercle stage was retained for estimation of *O. cumana* infestation (tubercles of different sizes were observed as illustrated in Fig. 1A), because at that stage it was possible to examine thoroughly the colonization of the root system. At later stages the quantitative assessment of infestation was made more complex by the presence of attached broomrapes at several different stages.

The level of broomrape infestation was examined five or six weeks post-inoculation by counting the total number of attached *O. cumana* tubercles on each plant. Because results of such experiments exhibited strong variability both within and between experiments (Fig. 1B), we performed six independent experiments to increase the total number of plants in each condition (AM-inoculated or not). The whole set of results was subsequently analyzed through a multi-way ANOVA, which allows to segregate the different sources of variation. Across the set of experiments, mycorrhizal plants were significantly more resistant to *O. cumana* (P = 0.008, ANOVA details in Table S1), although this effect was moderate (ca 20% less tubercles in average). The size of tubercles as estimated by their average dry weight was not significantly affected by mycorrhizal inoculation (15.4+/−1.7 mg in mycorrhizal plants *vs* 13.1+/−1.4 mg in non-mycorrhizal plants).

### Mycorrhizal Status Affects the Germination Stimulant Content of Root Exudates

Previous studies [22], [23] suggested that the protective effect of AM fungi against *Striga* infection involved a reduced production of germination stimulants. To investigate whether this was the case in the *O. cumana*/sunflower interaction, we assessed the germination of *O. cumana* seeds triggered by root exudates of mycorrhizal *vs* non-mycorrhizal plants. While both types of exudates could stimulate seed germination of *O. cumana*, this activity was markedly reduced in exudates of mycorrhizal plants: they induced only 35% germination of *O. cumana* seeds, *vs* 53% with non-mycorrhizal root exudates. The difference was significant (P<0.05) and suggested that root exudates of mycorrhizal sunflower plants may lack some germination stimulants present in non-mycorrhizal root exudates.

Strigolactones are recognized as a major class of germination stimulants for *Orobanche spp*, and have been detected in root exudates of a number of plants including sunflower [5], [34]. In this host plant, however, additional germination stimulants including DCL have been proposed to play an important role in the induction of *O. cumana* germination [6], [35]. Therefore, we tried to examine the strigolactone and DCL content of sunflower root exudates, in plants that were colonized or not by AM fungi.

Attempts were made to measure biochemically the abundance of these compounds. Analysis of the DCL content of root exudates by GC-MS/MS proved more sensitive than LC-MS/MS, but the amount of DCL remained close to the detection limit. Comparative analysis of mycorrhizal *vs* non-mycorrhizal root exudates did not reveal significant differences (data not shown).

Unfortunately, the sunflower strigolactones orobanchyl acetate and 5-deoxystrigol were undetectable in all root exudate extracts. The difficulties in detecting DCL and strigolactones may relate either to the sunflower variety used, or to the age of plants, both of which differ from those reported in the article describing biochemical analyses of DCL [6] and strigolactones [5]. In the present study it was necessary to wait for four weeks after AM inoculation to obtain significant root colonization. The plants may then have been too old for optimal production of germination stimulants.

**Figure 2:**
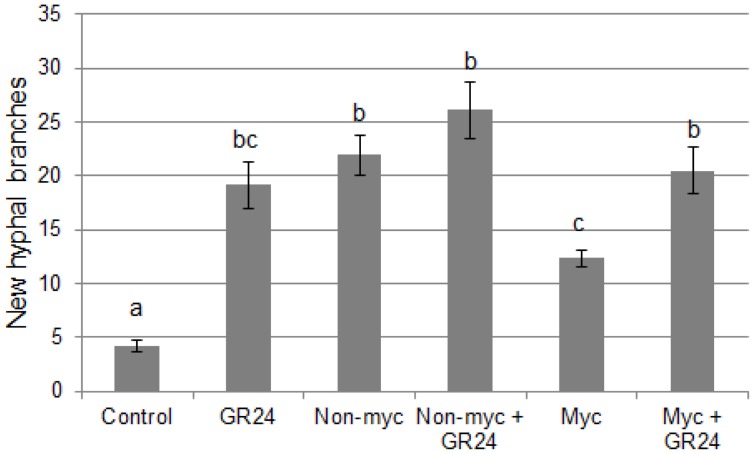
Investigation of the strigolactone content of root exudates. Ethyl acetate extracts of root exudates were dried and resuspended in 10% acetonitrile. Germinated spores of *G. rosea* were treated with root exudate extracts, and the newly formed hyphal apices were counted 48 h after treatment. Control: solvent only (10% acetonitrile). GR24∶10^−7^ M GR24 in 10% acetonitrile. Non-Myc and Myc: root exudates of non-mycorrhizal and mycorrhizal plants, respectively. Data represent the mean +/− s.e.m. obtained with 25 treated spores per treatment. Means were compared using one-way ANOVA followed by Tukey’s HSD test. Different letters indicate statistically significant differences between means (P<0.05).

The strigolactone content of root exudates was estimated using an AM fungus-based bioassay. The hyphal branching response of *G. rosea* germinated spores is highly sensitive to various strigolactones [19], [20]. Therefore, it allows an indirect comparison of the amount of strigolactones in different samples: the activity in root exudates of strigolactone-deficient mutants is strongly decreased in comparison with root exudates from wild-type plants [36]. In addition, DCL is not able to trigger such hyphal branching [37]. Root exudate extracts of non-mycorrhizal plants exhibited a strong activity in this bioassay (Fig. 2). This activity was markedly lower in root exudate extracts of mycorrhizal plants, and could be enhanced when the synthetic strigolactone analogue GR24 was added to these exudates, suggesting that their low activity was related to a low strigolactone content.

### Root Exudates of Mycorrhizal Plants have a Negative Impact on *O. cumana* Germination

**Figure 3:**
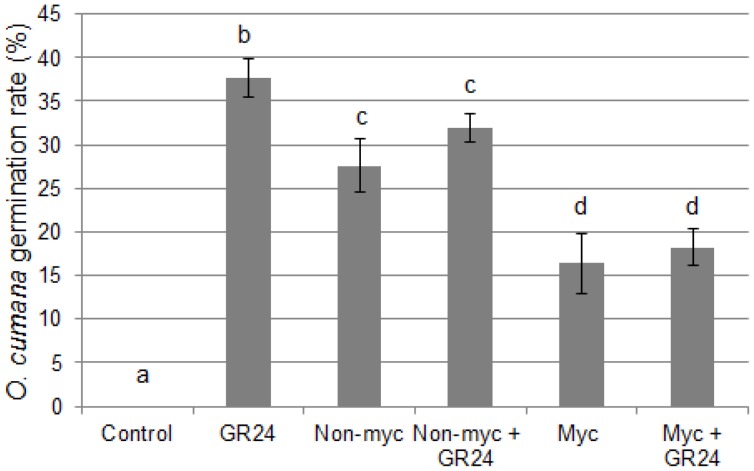
Induction of *O. cumana* germination by root exudates and GR24. Seeds were treated with GR24, and/or root exudates of mycorrhizal or non-mycorrhizal plants. Control: solvent only (0.1% acetone). GR24∶10^−8^ M GR24 in 0.1% acetone. Non-Myc and Myc: root exudates of non-mycorrhizal and mycorrhizal plants, respectively. Data represent the average germination percentage of five batches of approx. 100 seeds, +/−s.e.m. Means were compared using the Kruskal-Wallis test; different letters indicate statistically significant differences (P<0.05).

According to the above results, a reduced amount of germination stimulants could account for the reduced ability of root exudates of mycorrhizal plants to stimulate *O. cumana* germination. To further investigate this possibility, we added GR24 to sunflower root exudates, at a concentration of 10^−8^ M identical to that used for the positive control. As shown in Fig. 3, this did not improve the germination stimulant activity of mycorrhizal root exudates: it remained lower than that of non-mycorrhizal root exudates (complemented or not with GR24). This suggests that the lower germination stimulant activity of mycorrhizal root exudates is not only due to a reduced production of strigolactones by these roots.

Secondly, the germination rates obtained with root exudates (both mycorrhizal and non-mycorrhizal) complemented with GR24 were lower than that measured with GR24 alone (Fig. 3). Since these exudates were produced in nutrient solution, it cannot be completely ruled out that the salts contained in the solution contributed to the reduced germination rate. Nevertheless, mycorrhizal root exudates consistently led to lower germination rates as compared with non-mycorrhizal root exudates, establishing their stronger capacity to interfere with GR24-induced *O. cumana* germination.

### AM Fungal Spores and their Exudates Negatively Affect Broomrape Seed Germination

**Figure 4:**
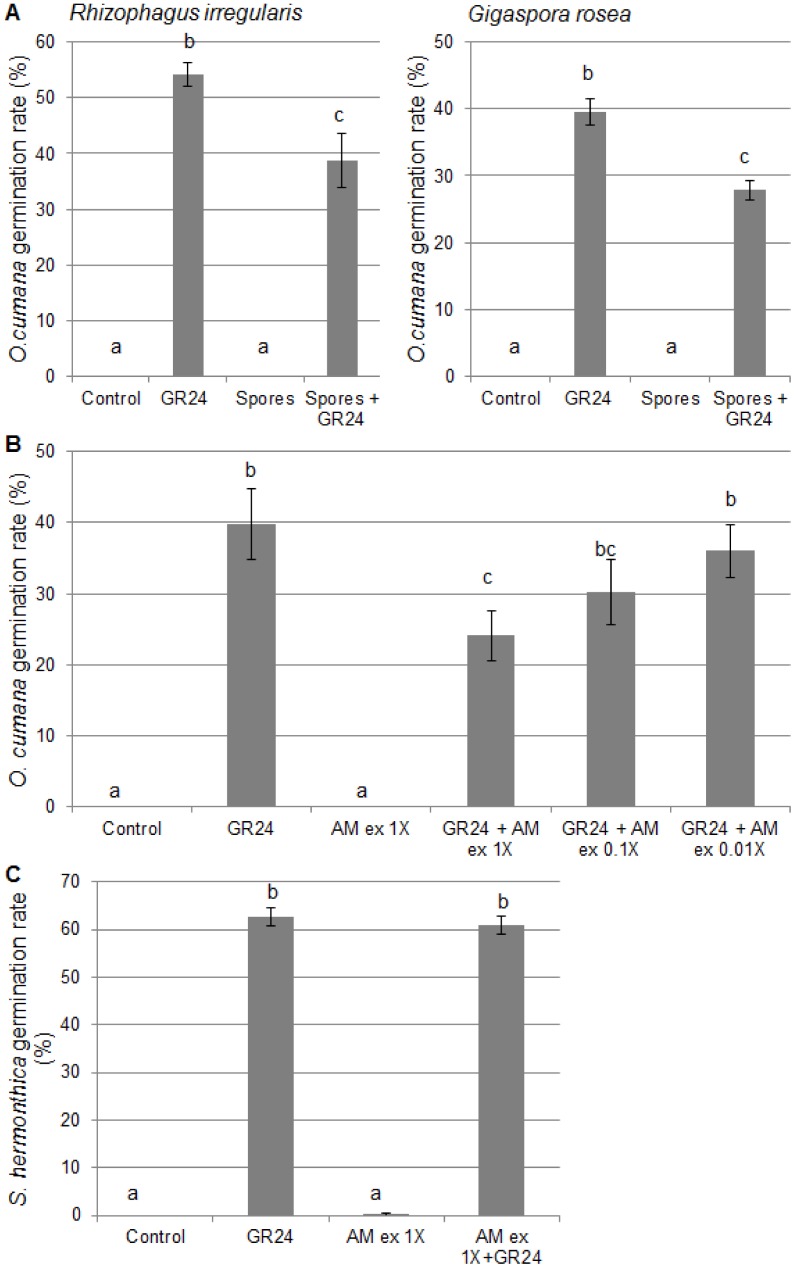
Germination of *O. cumana* and *S. hermonthica* in the presence of spores or spore exudates of AM fungi. A- *O. cumana* seed germination was induced by 10^−8^ M GR24 in the presence or absence of fungal spores of *R. irregularis* or *G. rosea*. Control: solvent only (0.1% acetone). B, C- Seeds of *O. cumana* (B) or *S. hermonthica* (C) were treated with 10^−8^ M GR 24 and/or *R. irregularis* spore exudates (AM ex) at different concentrations. 1× corresponds to 1,000 spores per mL. The “AM 1× exudate” negative control was complemented with 0.1% acetone in order to match the acetone concentration in the other samples. Data represent the average germination rate of five batches of approx. 100 seeds, +/− s.e.m. Means were compared using the Kruskal-Wallis test; different letters indicate significant differences (P<0.05).

The negative effect of mycorrhizal root exudates could be due to factors originating from the colonized roots, and/or from the fungus itself. Therefore, we next examined the effect of fungal spores or their exudates on *O. cumana* germination. With two phylogenetically distant AM fungi (*R. irregularis* and *G. rosea*), the seed germination rate was reduced in the presence of germinated fungal spores (Fig. 4A). Furthermore, fungus-free exudates of these spores could also exert a dose-dependent effect (Fig. 4B), which demonstrated that it is due to diffusible compounds rather than to volatile molecules or to direct contact between fungal structures and *O. cumana* seeds.

**Figure 5:**
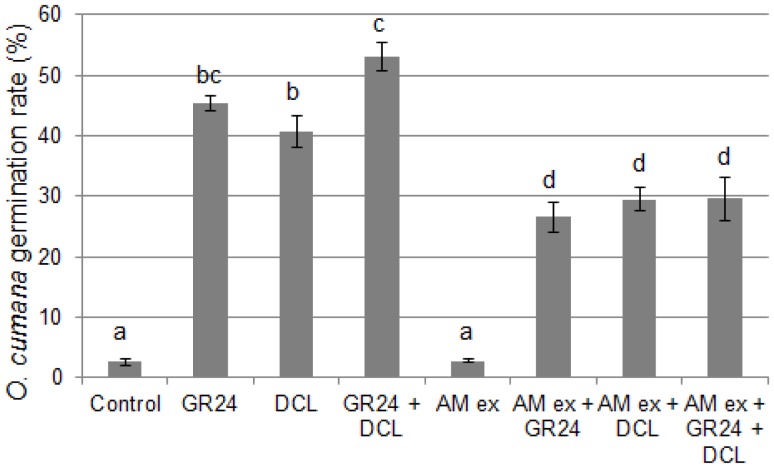
Response of *O. cumana* seeds to *R. irregularis* exudates and pure germination stimulants. AM ex corresponds to 1,000 spores per mL in 0.2% acetone. Control: solvent only (0.2% acetone). GR24 and DCL were used at a final concentration of 10^−8^ M in 0.2% acetone. Data represent the average germination rate of five batches of approx. 100 seeds, +/−s.e.m. Means were compared using the Kruskal-Wallis test. Different letters indicate significant differences (P<0.05).

The germination of *O. cumana* can be triggered with similar efficiencies by GR24 or DCL ([13], Fig. 5). We thus investigated whether AM fungal exudates could affect *O. cumana* germination regardless of the germination stimulant used. As shown in Fig. 5, the effect of AM spore exudates was the same when seeds were stimulated by GR24, DCL or both.

Various fungal toxins have previously been shown to inhibit parasitic plant seed germination [38]. This can be attributed either to toxicity or to effects on the germination process *per se*. To determine whether the effect of AM fungal exudates involved some degree of toxicity towards broomrape seeds, we measured the viability of *O. cumana* seeds after treatment with fungal exudates. No effect on seed viability could be detected using a TTC-based assay [31], regardless of the presence of GR24 (Table 1). In another experiment, seeds were incubated with 1X AM spore exudates for seven days, then washed in water to eliminate the exudates. Following exposure to GR24, the germination rate of these washed seeds was not significantly lower than that of seeds that had not been in contact with AM exudates (70.9%+/−3.9 *vs* 77.3%+/−4.6 respectively, P = 0.32). This indicated that AM spore exudates had not altered the seeds ability to germinate.

**Table 1:** Effect of crude exudates of *R. irregularis* on seed viability of *O. cumana*.

Treatment	Viability rate (%)	s.e.m.
Control (solvent only)	70.9	1.7
GR24	70.9	1.9
*R. irregularis* exudates 1X	70.3	1.5
*R. irregularis* exudates 1X+GR24	76.8	0.9

Seeds were stained with TTC and examined with a dissecting microscope. Data represent the average percentage of viable seeds. Five batches of approx. 100 seeds were used for each treatment. Means were compared using the Kruskal-Wallis test. No significant differences between treatments were found.

We then investigated whether this effect on seed germination could affect a wide range of plant seeds. Another broomrape species, *Phelipanche ramosa*, was first examined and results were very similar to those obtained with *O. cumana* (Fig. S1). In contrast, *Striga hermonthica* seed germination was not affected by AM spore exudates (Fig. 4C). Two non-parasitic dicot and monocot plant species, *Arabidopsis thaliana* and leek (*Allium porrum* L.) were also examined, and again their germination rate remained the same when treated with fungal exudates (Fig. S2).

## Discussion

Root exudates of mycorrhizal plants exhibited a clearly reduced ability to trigger *O. cumana* seed germination. This is in line with the previous studies of Lendzemo *et al*. [22] and Fernandez-Aparicio *et al*. [24] on sorghum and pea, respectively. The most immediate interpretation was that the production of the main germination stimulants, strigolactones and DCL, was affected by root colonization with AM fungi. Biochemical analyses of the DCL content of mycorrhizal *vs* non-mycorrhizal roots did not reveal significant differences, although these results should be taken with caution since in all samples the amount of DCL was very close to the detection limit. In contrast, an indirect bioassay for strigolactone activity (Fig. 2) suggested that the production or release of these compounds could be reduced in mycorrhizal plants, as has been observed in tomato [25]. Interestingly, however, this observation did not solely account for the decreased *O. cumana* germination in the presence of mycorrhizal root exudates. Rather, complementation experiments with GR24 demonstrated that mycorrhizal root exudates could reduce the germination of *O. cumana* triggered by GR24 (Fig. 3). It would be interesting to know if the sorghum and pea mycorrhizal roots previously analysed [22], [24] exhibited similar properties, and whether this could have contributed to the observed protective effects of AM fungi.

In addition to mycorrhizal roots, AM fungi living asymbiotically can also release factors that lead to a reduced germination of *O. cumana* (Fig. 4A and 4B). It remains unknown at this stage whether the same diffusible compounds are responsible for the observed effects of mycorrhizal root and fungal spore exudates on germination. Roots produce and secrete large numbers of molecules, many of which are active in the rhizosphere. For example, sunflower has been shown to produce phytoalexins that can inhibit *O. cumana* germination [39]. Such compounds could potentially contribute to the slight inhibitory effect noted with non-mycorrhizal root exudates, and possibly also to the stronger effect of mycorrhizal root exudates on seed germination, since mycorrhization is known to modify root metabolism [40]. In root exudates however, it is impossible to distinguish between compounds produced by the colonized root or by the fungus itself. In contrast, the analysis or fungal spore exudates allowed us to establish unequivocally that AM fungi alone can release diffusible compounds that interfere with GR24- or DCL-induced *O. cumana* germination. This observation shows that AM fungi can benefit their host plants before the symbiotic interaction is established.

Various hypotheses can be put forward to explain the effect of exudates from mycorrhizal roots or fungal spores on *O. cumana* germination. First, they could contain factors (such as enzymes or reactive oxygen species) that lead to the degradation of germination stimulants in the medium, or alternatively compounds that bind to the germination stimulants and prevent their action. Second, they could contain germination inhibitors (compounds that target germination *per se* or act as antagonists of germination stimulants). Two lines of evidence tend to argue against the first hypothesis, although they do not rule it out completely: a) addition of GR24 to mycorrhizal root exudates enhanced their hyphal branching activity (Fig. 2), indicating that GR24 was not significantly degraded nor sequestered in this experiment, but it should be born in mind that the kinetics here are different from a seed germination assay; and b) AM spore exudates did not affect the germination of *S. hermonthica* (Fig. 4C), again suggesting that sufficient active GR24 was available in the medium, but the minimal GR24 concentrations required to induce *S. hermonthica* and *O. cumana* germination may be different.

It would be interesting to test the effect of AM spore exudates on a larger number of plant species, especially parasitic, in order to assess the range of potential applications of our findings, and to help decipher the underlying mechanisms of action. No effect of AM exudates on the germination of non-parasitic plant species could be detected. This is not surprising since AM fungi live symbiotically with most land plants, and actually require the association with a living plant to complete their own life cycle [18].

Similar effects on seed germination were obtained with spores or spore exudates of two distant AM fungi. This suggests that these effects are a general property of *Glomeromycota*, and that a wide range of AM fungi could be used as biocontrol agents. Furthermore, the fact that mycorrhizal roots also exert such effects on *Orobanche* germination (Fig. 3) indicates that this protective effect of AM fungi could persist throughout the crop cycle. In addition to the use of AM inoculants, the biochemical identification of the active compound(s) may lead to novel opportunities to help keeping broomrapes under control.

The effect of mycorrhizal inoculation on root infestation (Fig. 1 and 3) was rather modest, as compared with some results of the studies on sorghum [21], [22]. In the sorghum studies however, the beneficial effects of AM fungi were strongly dependent on the host plant genotype, the tolerant variety being more responsive to the effect of mycorrhizae. The present study was carried out on a susceptible sunflower variety (2603), which may not be the most responsive to the protective effect of AM fungi.

It may seem surprising that the effects of AM exudates on *O. cumana* seed germination are stronger than those of AM inoculation on root infestation. On the other hand, only a small proportion of the seeds present in each pot (>1,000 seeds) develops to reach the tubercle stage (15 to 30 per pot after five to six weeks, Fig. 1). If one assumes a reasonable seed germination rate in these conditions (for example, a 20% germination rate would yield over 200 germinated *O. cumana* seedlings per pot), this suggests that various factors prevent the attachment of most seedlings to the host roots. This could explain why a reduced germination rate does not necessarily translate into a similarly reduced number of tubercles.

Various biocontrol agents have been tested against parasitic weeds. Among them, various fungi such as *Fusarium* and *Alternaria* spp. and *Myrothecium verrucaria* [41]–[43] have been used successfully against *Orobanche* and *Striga* spp. These fungi seem to act differently from AM fungi: they behave as pathogens of the parasite, infecting its tissues or producing toxins [38], [42], [44], [45]. Mycorrhizal fungi offer two advantages as biocontrol agents: they are not pathogenic to any crop plant, and provide important additional benefits such as an improved water and mineral nutrition. In some cases they can also confer a protection against fungal pathogens, such as *Plasmopara helianthii* for sunflower [46]. They can adapt to a wide range of environments and stay in the soil for many years after inoculation. Therefore, their use as biocontrol agents offers an attractive complementary approach to breeding strategies and other biocontrol methods.

## Supporting Information

Figure S1 **Germination of** ***P. ramosa*** **seeds in the presence of spore exudates of AM fungi.** *P. ramosa* seed germination was induced by GR24 in the presence or absence of *R. irregularis* spore exudates. Control: solvent only (0.1% acetone). GR24∶ 10^−8^ M GR24 in 0.1% acetone. Spore exudates (AM ex) were used at different concentrations; 1× corresponds to 1,000 spores per mL. Data represent the average germination percentage of five batches of approx. 100 seeds, +/−s.e.m. Means were compared using the Kruskal-Wallis test; different letters indicate significant differences (P<0.05).
(EPS)

Figure S2 **Effect of AM spore exudates on seed germination of non-parasitic plant species.** The germination rates of *Arabidopsis thaliana* (A) and leek seeds (B) were measured in the presence or absence of *R. irregularis* spore exudates (AM ex 1X, obtained with 1,000 spores per mL). Six to twelve batches of 50 seeds were tested, values represent the mean +/− s.e.m. Means were compared using Student’s t-test and no significant differences were found (P = 0.79 for *A. thaliana* and P = 0.89 for leek).
(EPS)

Table S1 **Multi-way ANOVA analysis of experiments described in** **Figure 1** **.**
(DOCX)
