# Supplementary material for: Reduced Germination of Orobanche cumana Seeds in the Presence of Arbuscular Mycorrhizal Fungi or Their Exudates
Source: PLoS One. 2012 Nov 7;7(11):e49273. doi: 10.1371/journal.pone.0049273 (PMC3492269; doi:10.1371/journal.pone.0049273)
Supplement: Table S1 — Multi-way ANOVA analysis of experiments described in Figure 1 . (DOCX) [file pone.0049273.s003.docx]

### Supporting Table S1. Multi-way ANOVA analysis of experiments described in Figure 1.

|  | Degrees of freedom | Sum of squares | F value | P value |
| --- | --- | --- | --- | --- |
| Treatment | 1 | 334 | 7.332 | 0.008007 |
| Experiment | 4 | 2470.5 | 13.5598 | 8.116 e-09 |
| Duration of culture | 1 | 769.8 | 16.8999 | 8.25 e-05 |
| Duration of culture: treatment | 1 | 8.5 | 0.1864 | 0.666908 |
| Experiment:Treatment | 4 | 94.8 | 0.5204 | 0.720927 |
| Residuals | 97 | 4418.1 |  |  |

The first column shows the factors taken into account: treatment (AM-inoculated or not), experiment, duration of experiment (time of harvest = 5 or 6 weeks post inoculation), and interactions between these factors. P values indicate a highly significant effect of mycorrhizal inoculation, of duration of culture as well as highly significant differences between independent experiments. No interaction between these factors could be detected.
